# fNIRS Evidence for Recognizably Different Positive Emotions

**DOI:** 10.3389/fnhum.2019.00120

**Published:** 2019-04-09

**Authors:** Xin Hu, Chu Zhuang, Fei Wang, Yong-Jin Liu, Chang-Hwan Im, Dan Zhang

**Affiliations:** ^1^Department of Psychology, Tsinghua University, Beijing, China; ^2^Department of Computer Science and Technology, Tsinghua University, Beijing, China; ^3^Department of Biomedical Engineering, Hanyang University, Seoul, South Korea

**Keywords:** positive emotion, fNIRS, oxy-hemoglobin, deoxy-hemoglobin, classification

## Abstract

The behavioral differentiation of positive emotions has recently been studied in terms of their discrete adaptive functions or appraising profiles. Some preliminary neurophysiological evidences have been found with electroencephalography or autonomic nervous system measurements such as heart rate, skin conductance, etc. However, the brain’s hemodynamic responses to different positive emotions remain largely unknown. In the present study, the functional near-infrared spectroscopy (fNIRS) technique was employed. With this tool, we for the first time reported recognizable discrete positive emotions using fNIRS signals. Thirteen participants watched 30 emotional video clips to elicit 10 typical kinds of positive emotions (joy, gratitude, serenity, interest, hope, pride, amusement, inspiration, awe, and love), and their frontal neural activities were simultaneously recorded with a 24-channel fNIRS system. The multidimensional scaling analysis of participants’ subjective ratings on these 10 positive emotions revealed three distinct clusters, which could be interpreted as “playfulness” for amusement, joy, interest, “encouragement” for awe, gratitude, hope, inspiration, pride, and “harmony” for love, serenity. Hemodynamic responses to these three positive emotion clusters showed distinct patterns, and HbO-based individual-level binary classifications between them achieved an averaged accuracy of 73.79 ± 11.49% (77.56 ± 7.39% for encouragement vs. harmony, 73.29 ± 11.87% for playfulness vs. harmony, 70.51 ± 13.96% for encouragement vs. harmony). Benefited from fNIRS’s high portability, low running cost and the relative robustness against motion and electrical artifacts, our findings provided support for implementing a more fine-grained emotion recognition system with subdivided positive emotion categories.

## Introduction

Feeling proud of good grades, getting amused by funny jokes, or being peaceful when staying with families…There is more than one kind of “happiness” in our daily life but the diversity of those positive emotions was much understated in previous studies. Positive emotions were often treated as a homogeneous group to be compared with negative or neutral emotion states, and the differentiation within positive emotions was largely neglected. More recently, some theoretical frameworks of discrete positive emotions have been proposed, mainly from the appraisal or adaptive function perspectives (Goetz et al., 2010; Fredrickson, 2013; Shiota et al., 2014; Tong, 2015; Graham et al., 2017). The appraisal approach to differentiate positive emotions could trace back to Smith and Ellsworth (1985) and Ellsworth and Smith (1988), which proposed six pleasant feeling states (hope/confidence, love, playfulness, tranquility, challenge, interest) associated with distinct appraisal patterns (appraisal of effort, agency, and certainty). Goetz et al. (2010) examined another three positive emotions (enjoyment, pride, contentment) and different relations with cognitive appraisal antecedents were found in the perception of control and value. Tong (2015) differentiated 13 positive emotions (amusement, awe, challenge, compassion, contentment, gratitude, hope, interest, joy, pride, relief, romantic love, and serenity) in 13 appraisal dimensions (pleasantness, relevance, problem, etc.), and the accuracies to classify positive emotions with appraisal profiles were reported to be above chance levels. Meanwhile, another vein of research focuses on the adaptive function of positive emotions. For example, Shiota et al. (2014) proposed a functional framework to define a number of discrete emotions, which included pride, amusement, nurturant love, attachment love, contentment, enthusiasm, awe, and sexual desire. Enthusiasm and contentment were thought as responses to material opportunities; Sexual desire, attachment love, nurturant love, as well as pride, were considered adaptive for their implications in the social domain; Amusement and awe were suggested to reflect opportunities to learn. Fredrickson (2013) qualitatively described both the appraisal themes and functional resources accrued of 10 representative positive emotions (joy, gratitude, serenity, interest, hope, pride, amusement, inspiration, awe, love), but empirical evidence is still limited. Most of those studies built their theories either on participants’ subjective appraisals or researchers’ personal interpretations for the proposed emotions; nevertheless, it is worth noting that cognitive appraisals do not necessarily cover all the aspects of the subjective experience of emotions. More importantly, the basic components of human emotion consist of not only subjective experience, but also behavioral expressions and neural responses (Izard, 1977). In the following paragraphs, we’ll briefly review the empirical evidence for the differentiation of positive emotions based on these components.

### Subjective Experience

Evidence for the differentiated subjective experiences of positive emotions has been found both from people’s reports of the induced emotion states and daily emotion experiences. Recently, Linley et al. (2016) collected people’s retrospective recall of the frequency and intensity of positive emotion experience in their daily life, and provided support for a set of 50 discrete positive emotions. Another study by Cowen and Keltner (2017) used 2,185 short video clips to elicit emotion responses from 853 participants and found 27 distinct varieties of the reported emotion experiences. About half of these 27 distinct varieties were positive emotions, and evidences for the difference between nuanced positive emotions such as romantic love and sexual desire were also reported.

### Behavioral Expressions

Campos et al. (2013) found evidence for the different patterns of eight positive emotions in facial expression and body movement. Sauter (2017) reviewed the literature on the non-verbal expressions of positive emotions and reported the distinct recognizable vocal or facial displays of six positive emotions (amusement, awe, interest, and relief, pride, and elation). Hofmann et al. (2017) examined the Duchenne Displays of 16 positive emotions proposed by Ekman (2003), and found they differed in the intensity of Duchenne Displays and the propensity to induce laughter. While these studies were mostly based on static facial features or body gestures, review Mortillaro and Duk’es (2018) also stressed the importance of the dynamic facial expressions and body movement to differentiate positive emotions.

### Neural Responses

Kreibig (2010) reviewed studies on the autonomic nervous system (ANS) responses to positive emotions, and reported considerable ANS specificity of eight distinct positive emotions. Shiota et al. (2011) assessed people’s sympathetic and parasympathetic activations to five kinds of positive emotions, and provided evidence for the existence of physiologically distinct positive emotions in the aspect of ANS respondence. In addition to these ANS findings, a few studies have explored the central nervous system (CNS) responses to different positive emotions, mainly using the electroencephalography (EEG) technique. By analyzing the EEG-based neural electrical activities, Liu et al. (2017) realized a real-time recognition of three positive emotions (joy, amusement, and tenderness); in our previous EEG study, 10 typical positive emotions were included and recognizable EEG patterns of three distinct positive emotion clusters were found (Hu et al., 2017).

However, direct evidence of hemodynamic responses for the differentiation of positive emotions is still absent. Hemodynamic responses reflect the blood oxygenation level in the brain. It has been generally accepted that hemodynamic and neural electrical signals (e.g., EEG) provide complementary information about the underlying neural mechanism in various cognitive functions (Debener et al., 2006; Balconi et al., 2015). Using functional magnetic resonance imaging (fMRI) or functional near-infrared spectroscopy (fNIRS) technique, many researches have reported recognizable hemodynamic responses between positive versus negative emotions (eg., Tai and Chau, 2009; Sitaram et al., 2010; Moghimi et al., 2012; Bush et al., 2018) but not between different positive emotions. Nevertheless, the findings of the general-level positive emotion (usually termed “happiness”) have been mixed (Vytal and Hamann, 2010; Hamann, 2012; Bendall et al., 2016). For instance, one study found positive emotions was accompanied by lower HbO responses in the right PFC compared to negative emotions (Balconi et al., 2015). Yet another study reported positive emotion was associated with increased oxygenation (HbO-HbR) in medial rostral PFC compared to negative states (Kreplin and Fairclough, 2013). Such inconsistent results might be explained by the oversimplified categorization of positive emotions and different manipulations for “positive” in different researches. Indeed, recent fMRI studies have also investigated the neural mechanisms of a few positive emotions and distinct brain regions were found for different positive emotions: amusement was found in relation to the activation in the left amygdala (Bartolo et al., 2006); gratitude was reported to be correlated with brain activity in the anterior cingulate cortex and medial prefrontal cortex (PFC) (Fox et al., 2015); Pride was found associated with activations in the right posterior superior temporal sulcus and left temporal pole, while joy activated the ventral striatum and insula/operculum (Takahashi et al., 2008). Employing fNIRS technique, researchers have also obtained a few preliminary findings: esthetic positive emotion was associated with activation in medial rostro PFC (Kreplin and Fairclough, 2013); Maternal and grandmaternal love was found involved with right and anterior PFC (Nishitani et al., 2011; Kida et al., 2014). Taken together, as different positive emotions seemed to be associated with distinct brain regions, these studies support the plausibility of differentiating positive emotions with hemodynamic responses. However, as most of these studies included only one positive emotion per study, it is necessary to include multiple positive emotions in a single study, in order to provide direct hemodynamic evidence for a more comprehensive and complete overview of the neural mechanisms of different positive emotions.

Compared to fMRI, fNIRS is less restrictive, more comfortable, and portable. This advantage may be especially crucial for positive emotion research, as unwanted interferences from device noises and claustrophobic environment are avoided, allowing for more natural positive emotion experience. Therefore, the present study employed fNIRS to study different positive emotions. We empirically focused on the PFC, because it involves less motion artifacts and signal attenuation due to hairs (Naseer and Hong, 2015), and previous emotion related fNIRS studies have reported positive findings in the PFC region, both for those researches focused on the general emotion valence (e.g., Tai and Chau, 2009; Moghimi et al., 2012; Trambaiolli et al., 2018; Wang et al., 2018) and specific positive emotions (e.g., Nishitani et al., 2011; Kreplin and Fairclough, 2013; Kida et al., 2014). Following our previous study (Hu et al., 2017), emotional videos were used to elicit 10 kinds of positive emotions (joy, gratitude, serenity, interest, hope, pride, amusement, inspiration, awe, and love), and the corresponding hemodynamic signals were recorded with an fNIRS system.

The aim of the present study is to explore the hemodynamic activities of different positive emotions. We hypothesize to observe distinct hemodynamic responses associated with different positive emotions. Here we referenced to existing fNIRS based affective computing studies (e.g., Tai and Chau, 2009; Moghimi et al., 2012; Heger et al., 2013; Aranyi et al., 2016), used a machine learning approach at a single-participant level: the classification accuracies of the fNIRS responses elicited by different positive emotion videos were taken to reflect the neural differentiation of positive emotions at the hemodynamic level. The classifications between positive and negative emotions were conducted as well, to provide a baseline for evaluation the results from between positive emotions. Such an approach could provide neural evidence not only for a better understanding of different positive emotions, but also for implementing practical brain-computer interface systems for emotion state recognition.

## Materials and Methods

### Participants

Fifteen college students (seven females, mean age: 22.5 years, ranging: 20–25 years) participated in the experiment as paid volunteers. All participants had normal hearing, normal or corrected-to-normal vision. Written informed consent was obtained from all participants. The study was conducted in accordance with the Declaration of Helsinki and approved by the local Ethics Committee of Tsinghua University. Data from two participants was discarded, due to technical problems during data recording.

### Materials

Thirty clips of films were used to elicit 10 typical kinds of positive emotions (joy, gratitude, serenity, interest, hope, pride, amusement, inspiration, awe, and love, following the proposal in Fredrickson, 2013). One neutral and six negative emotion stimuli (half with high arousal and the other half with low arousal) were used as a control condition. All the stimuli in use were the same as those in our previous study (Hu et al., 2017), except that one film clip to induce pride (the publicity film of Tsinghua University) was replaced by a TV news about the launching of Shenzhou-10 spacecraft for the general Chinese audience. The average duration of all the stimuli was 70 s (varied between 30 to 129 s). For those film clips containing non-Chinese dialogues, Chinese subtitles were added to guarantee a full understanding of the contents. Detailed information of the materials can be found in the Supplementary Table S1.

### Procedure

The participants watched all the 37 film clips (30 positive, 6 negative, and 1 neutral) on an LCD monitor (22-inch, 60 Hz refreshing rate) in a laboratory environment, with their hemodynamic activities simultaneously recorded. After watching each film clip, participants were asked to report their emotion states on the 10 positive emotions and another four emotion dimensions (arousal, valence, familiarity, and liking) on seven-point Likert scales (1 = not at all, 7 = extremely). Between two sequential trials, participants took a rest for at least 45 s to recover from the previously induced emotion state.

Prior to the experiment, the participants were given an explanation of the 14 emotional items and the experimental procedure. Then, two practice trials were performed to get the participants familiarized with the procedure. In the formal experiment, the neutral clip was first presented, followed by the six negative emotion clips, and then the 30 positive emotion clips. The orders of the clip presentations were randomized within the negative and positive emotion clips, respectively. The experiment procedure was programmed in MATLAB (The Mathworks, United States) using the Psychophysics Toolbox 3.0 extensions (Brainard, 1997).

### fNIRS Recordings

**FIGURE 1:**
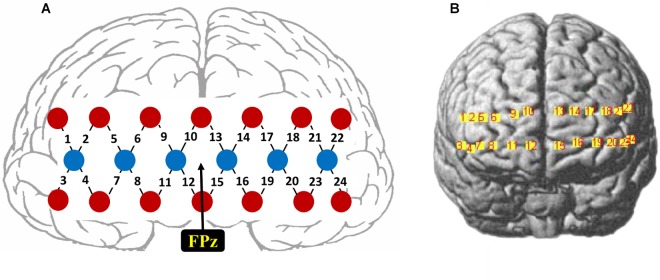
**(A)** Schematic illustration of the fNIRS probe layout. **(B)** The topographical distribution of the fNIRS recording channels.

The fNIRS signals were recorded using a 24-channel fNIRS system (NirScan, HuiChuang, China) at a sampling rate of 50 Hz. Near-infrared light of three different wavelengths (785, 808, and 850 nm) was used to detect the concentration change of oxy-hemoglobin (HbO) and deoxy-hemoglobin (HbR). Twenty probes (6 sources and 14 detectors) were placed to cover the frontal cortex (between-probe distance of 30 mm), resulting in a total of 24 channels, as shown in Figure 1A. The center of the middle probe set row was placed approximately at FPz, according to the 10/20 international system. The topographical distribution of the fNIRS channels were visualized on the standard human cortex surface using the NirsScan software (Figure 1B).

### Data Analysis

#### Behavioral Data Analysis

Intra-class correlation coefficients (ICC) were calculated for all the 14 emotion items to examine the reliability of the ratings across participants. Then, repeated measures analysis of variance (rmANOVA) and *post hoc* paired *t*-tests were employed to check whether these film clips elicited the expected emotions. For each group of three film clips designated to elicit one specific positive emotion, participants’ ratings on the 10 positive emotion items were compared to see whether the target emotion was more prominent than the other emotions. The false discovery rate (FDR) method was used to correct the *p*-values from the *post hoc t*-tests (Benjamini and Hochberg, 1995).

To obtain a general overview of the relationships among the 10 positive emotions, Pearson correlation coefficients were calculated on the cross-participant averaged ratings per film clip, for every pair of the 10 positive emotions. As significant correlations were found among many pairs of positive emotions (see Results), we then applied unsupervised multidimensional scaling (MDS) method on the cross-participant average ratings of the 30 positive emotion film clips, to further characterize the similarity across all the 10 positive emotions. These 10 positive emotions were manually categorized into three clusters according to their geometric similarity in the MDS space, and each film clip’s cluster scores were calculated by averaging the ratings of all the emotions within each cluster, resulting in three cluster scores. For example, emotions in the “playfulness” cluster are interest, joy, and amusement. For a certain film clip, if the ratings of those three emotions were 1, 2, 2, then the “playfulness” score for this film clip would be 1.67. And the other two cluster scores were calculated in the same way.

#### fNIRS Signal Preprocessing

Raw light intensity data was filtered by 0.01–0.2 Hz bandpass to remove common noises including the physiological noises due to heartbeats, respirations, and Mayer waves (Zhang et al., 2017; Trambaiolli et al., 2018; Wang et al., 2018). Then, the filtered signals were converted into relative concentration changes of HbO and HbR according to the modified Beer-Lambert law (Scholkmann et al., 2014).

Specifically, the modified Beer-Lamber Law is formated as follows,

(1) $$\begin{array}{cc} {\mathrm{OD}}^{{\text{λ}}_{\text{i}}}=\ln\frac{I_{\text{oi}}}{I_{\text{I}}}=({\text{ε}}_{\text{HbO}}^{{\text{λ}}_{\text{i}}}C_{\text{HbO}}+{\text{ε}}_{\text{HbR}}^{{\text{λ}}_{\text{i}}}C_{\text{HbR}})\times r\times {\mathrm{DPF}}^{{\text{λ}}_{\text{i}}} & i=1,\;2,\;3 \end{array}$$

(2) $$\begin{array}{cc} \Delta {\mathrm{OD}}^{{\text{λ}}_{\text{i}}}=({\text{ε}}_{\text{HbO}}^{{\text{λ}}_{\text{i}}}\Delta C_{\text{HbO}}+{\text{ε}}_{\text{HbR}}^{{\text{λ}}_{\text{i}}}\Delta C_{\text{HbR}})\times r\times {\mathrm{DPF}}^{{\text{λ}}_{\text{i}}} & i=1,\;2,\;3 \end{array}$$

where the variable _ε_ is the wavelength-dependent extinction coefficient for each hemoglobin types. The *DPF* (differential path-length factor) is added to account for the true effective path length between source and detector and *r* represents the linear distance of the paired probes. The change in light absorption, referred to as delta optical density, Δ*OD*. Δ*C*_HbO_ and Δ*C*_HbR_ represent the relative concentration changes of HbO and HbR respectively. HbO and HbR can be calculated by the following equation,

(3) $$\left(\begin{array}{c} \Delta C_{\text{HbO}}\\ \Delta C_{\text{HbR}} \end{array}\right){\left(\begin{array}{ll} {\text{ε}}_{\text{HbO}}^{{\text{λ}}_{1}} & {\text{ε}}_{\text{HbR}}^{{\text{λ}}_{1}}\\ {\text{ε}}_{\text{HbO}}^{\text{λ}2} & {\text{ε}}_{\text{HbR}}^{\text{λ}2}\\ {\text{ε}}_{\text{HbR}}^{\text{λ}3} & {\text{ε}}_{\text{HbO}}^{\text{λ}3} \end{array}\right)}^{-1}\left(\begin{array}{l} \Delta {\mathrm{OD}}^{{\text{λ}}_{1}}/(r\times {\mathrm{DPF}}^{{\text{λ}}_{1}})\\ \Delta {\mathrm{OD}}^{{\text{λ}}_{2}}/(r\times {\mathrm{DPF}}^{{\text{λ}}_{2}})\\ \Delta {\mathrm{OD}}^{{\text{λ}}_{3}}/(r\times {\mathrm{DPF}}^{{\text{λ}}_{3}}) \end{array}\right)$$

HbO and HbR responses to each film clip were baseline corrected by subtracting the average response from the 10-s time window before the presentation of the film clip, then signals corresponding to the last 30 s of each film clip were extracted in order to obtain maximal emotional responses (following the procedure in Koelstra et al., 2012), and cut into three non-overlapping 10-s samples for further analyses. Besides band-pass filtering, we did not perform additional artifact rejection procedures.

#### Correlation Analysis

The response patterns to different positive emotion clusters were characterized by computing the Pearson correlation between the HbO/HbR responses of each individual fNIRS channel and three positive emotion scores. The correlation between HbO/HbR responses and the emotion valence ratings were also computed. The correlations were calculated for each individual participant, and the data of only positive emotion film clips was used. The topographies of the across-participant average Pearson correlation coefficients between the rating of emotion scores and the HbO/HbR responses were expected to illustrate the neural responses to the three positive emotion clusters.

#### Classification Based Analysis

A classification-based approach was adopted to evaluate the neural differentiation of different positive emotion clusters. A series of binary classifications were employed between (a) each positive emotion cluster and negative emotion, and (b) all the pairs of the three positive clusters. To be noted, there are thirty positive emotion stimuli and only six negative emotion stimuli in total. To have balanced sample sizes for the above classifications and to allow a direct comparison of these classification results, for each positive emotion cluster, six film clips with the highest cluster scores were selected, since there were six film clips for the negative emotion condition. The features for classifications were the HbO or HbR responses from all the 24 fNIRS channels, leading to 24 feature dimensions representing the spatial response patterns. These features were calculated as the average responses over each 10-s sample from the extracted 30-s period per each film clip, resulting in 3 (samples per film) × 6 (films per category) = 18 samples for each emotion category per participant. The linear-kernel-based support vector machine (SVM) classification method was employed, using the function provided by the Statistics and Machine Learning Toolbox of Matlab. The classifications were carried out using either HbO or HbR features separately, and both the HbO and HbR based classification were conducted on the basis of each individual participant’s data.

The pairwise binary classifications of the three positive emotion clusters were expected to indicate the separability of different positive emotions on the hemodynamic level, and the classifications between each positive emotion cluster and negative emotion served as a comparison. To calculate the chance level of these classifications, permutation tests were conducted by randomly shuffling the labels 100 times for each classifier, and the mean of these 100 shuffle-based accuracies determined the chance levels. All the reported results were based on sixfold cross-validations. The selection of sixfold is for a convenience purpose: there were 18 + 18 = 36 samples per binary classification, hereby each fold had six samples for testing.

## Results

Emotion ratings from different participants showed strong consistency, as revealed by the ICCs of all the ratings on the 14 emotion items. ICC values varied from 0.72 (serenity) to 0.95 (amusement), with a mean ICC of 0.91 (*SD* = 0.06), indicating good reliabilities across the participants.

**FIGURE 2:**
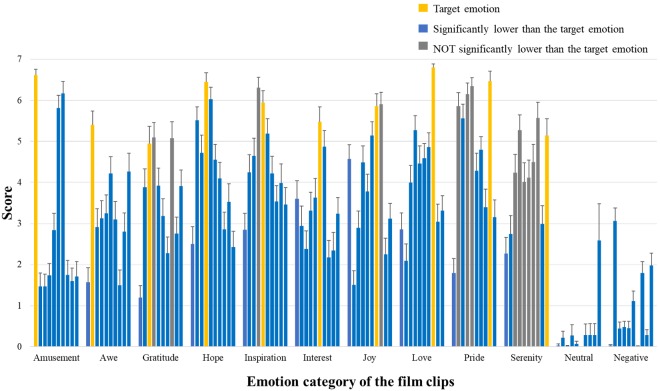
Basic information of emotion ratings. Participants’ ratings on the 10 positive emotional experience items. For each emotion category of film clips, the 10 bars indicate the mean ratings of these clips on amusement, awe, gratitude, hope, inspiration, interest, joy, love, pride, and serenity (from left to right). The bars in yellow show the ratings on the corresponding target emotion; The bars in blue mean their ratings significantly lower than those of the target emotion (*post hoc* paired *t*-tests *p* < 0.05, FDR corrected); The bars in gray indicate ratings NOT significantly different from those of the target emotion (*p* > 0.05, FDR corrected).

More than half of the film clips indeed showed the highest ratings on the target emotion (rmANOVA *p* < 0.001 for all positive emotion items and *post hoc* paired *t*-tests showed significantly higher ratings for the target emotion, *p* < 0.05) (Figure 2). However, as the correlation analyses showed, some positive emotions are highly correlated, and in some cases the target emotions did not significantly differ from other similar emotions (e.g., for film clips designated to elicit “pride,” ratings on “hope,” “inspiration,” and “awe” did not significantly differ from the ratings on “pride”) or even lower than them (e.g., for film clips designated to elicit “inspiration,” ratings on “inspiration” are actually lower than “hope”).

**FIGURE 3:**
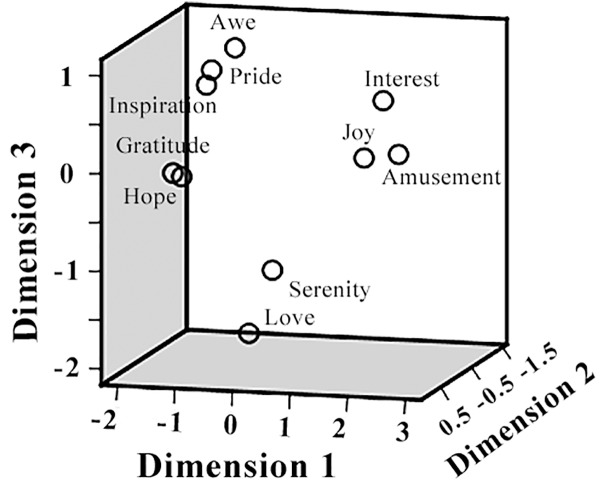
Multidimensional scaling (MDS) space of 10 positive emotions. The MDS space shows the similarity of the 10 positive emotions, based on the participants’ subjective ratings.

The pairwise correlation coefficients between the ratings on different positive emotions as well as arousal, valence, liking, and familiarity, were listed in Table 1. Significant correlations were observed in many cases. For example, participants’ ratings on “inspiration” and “hope” achieved a correlation coefficient of 0.94 (*p* < 0.05), indicating a considerable overlap between the feelings of inspiration and hope in the present film clip materials. The follow-up MDS analysis revealed a clear separation of these positive emotions in three clusters, resembling the behavioral results in our previous EEG study (Kruskal’s Stress I = 0.074, Figure 3). Cluster-1 is composed of awe, gratitude, hope, inspiration and pride, which is interpreted as “encouragement”; Cluster-2 is constituted by amusement, interest and joy, which is interpreted as “playfulness”; and Cluster-3 consists of love and serenity, which is interpreted as “harmony.”

**Table 1:** Pairwise correlation coefficients between the 14 emotion items.

	Amusement	Awe	Gratitude	Hope	Inspiration	Interest	Joy	Love	Pride	Serenity	Arousal	Valence	Familiarity	Liking
Amusement	–													
Awe	−0.48^∗∗^	–												
Gratitude	−0.05	0.56^∗∗^	–											
Hope	0.12	0.44^∗∗^	0.94^∗∗^	–										
Inspiration	0.27	0.47^∗∗^	0.87^∗∗^	0.94 ^∗∗^	–									
Interest	0.81^∗∗^	−0.05	0.36^∗^	0.51^∗∗^	0.66^∗∗^	–								
Joy	0.88^∗∗^	−0.23	0.31^∗^	0.48^∗∗^	0.60^∗∗^	0.92^∗∗^	–							
Love	0.14	−0.14	0.51^∗∗^	0.59^∗∗^	0.41^∗∗^	0.26	0.38^∗^	–						
Pride	0.03	0.62^∗∗^	0.85^∗∗^	0.83^∗∗^	0.89^∗∗^	0.45^∗∗^	0.41^∗∗^	0.34^∗^	–					
Serenity	−0.20	0.30	0.50^∗∗^	0.46^∗∗^	0.30	0.11	0.06	0.45^∗∗^	0.37^∗^	–				
Arousal	0.62^∗∗^	−0.02	0.09	0.19	0.42^∗∗^	0.56^∗∗^	0.58^∗∗^	−0.11	0.31	−0.56^∗∗^	–			
Valence	0.63^∗∗^	0.07	0.68^∗∗^	0.79^∗∗^	0.85^∗∗^	0.85^∗∗^	0.86^∗∗^	0.50^∗∗^	0.68^∗∗^	0.31^∗^	0.42^∗∗^	–		
Familiarity	0.43^∗∗^	0.02	0.33^∗^	0.41^∗∗^	0.53^∗∗^	0.51^∗∗^	0.53^∗∗^	−0.02	0.48^∗∗^	−0.02	0.50^∗∗^	0.58 ^∗∗^	–	
Liking	0.69^∗∗^	0.06	0.59^∗∗^	0.71^∗∗^	0.81^∗∗^	0.92^∗∗^	0.89^∗∗^	0.41^∗∗^	0.64^∗∗^	0.20	0.53^∗∗^	0.94^∗∗^	0.58^∗∗^	–

*^∗^p < 0.05, ^∗∗^p < 0.01.*

**FIGURE 4:**
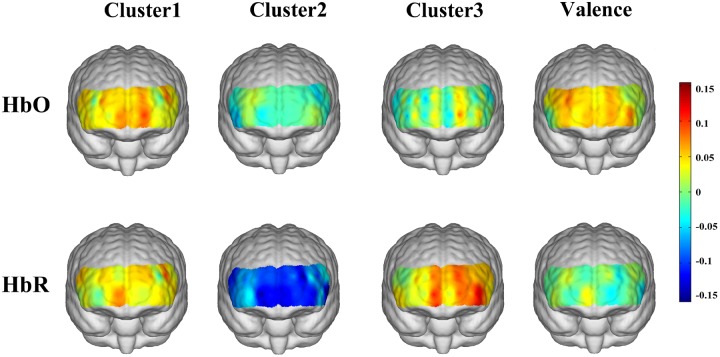
Correlations between emotion scores and HbO/HbR responses.

Figure 4 shows the topographies of the cross-participants averaged correlation coefficients between the HbO/HbR responses and the corresponding emotion scores (three positive emotion clusters and emotion valence). For the “encouragement” emotions (awe, gratitude, hope, inspiration, and pride), positive correlations with HbO responses were observed over the medial prefrontal area, and a mild right lateral pattern was found for HbR responses. The “playfulness” emotions (amusement, interest, and joy) showed prominent negative correlations with HbR responses over the whole frontal area. The “harmony” emotions (love and serenity) were associated with left lateral frontal activations both for HbO and HbR. The general emotion valence also showed mild lateral activations for HbO but not for HbR. However, none of these correlation results were significant after FDR correction.

The HbO-based binary classification accuracies for each positive emotion cluster vs. negative emotion are shown in Table 2. Individual-level binary classifications among them achieved an averaged accuracy of 83.69 ± 9.19% (83.33 ± 8.56% for encouragement vs. negative, 82.48 ± 10.17% for playfulness vs. negative, 85.26 ± 9.31% for harmony vs. negative; The chance levels determined by permutation tests were 49.29 ± 13.60, 49.06 ± 13.53, 49.08 ± 13.48%, respectively). The HbR-based results are shown in Table 3. Individual-level binary classifications among them achieved an averaged accuracy of 79.06 ± 9.47% (80.34 ± 9.98% for encouragement vs. negative, 80.34 ± 7.21% for playfulness vs. negative, 76.50 ± 11.03% for harmony vs. negative; The chance levels determined by permutation tests were 48.86 ± 12.85, 48.37 ± 13.05, 48.55 ± 12.72%, respectively).

**Table 2:** HbO-based binary classification accuracies between negative emotion and each positive emotion cluster.

Participant	Accuracy (%)		
	Negative vs. Cluster 1	Negative vs. Cluster 2	Negative vs. Cluster 3
1	69.44	86.11	77.78
2	86.11	91.67	88.89
3	86.11	72.22	72.22
4	91.67	94.44	97.22
5	97.22	63.89	83.33
6	75.00	66.67	94.44
7	91.67	77.78	80.56
8	72.22	83.33	94.44
9	72.22	91.67	75.00
10	86.11	94.44	97.22
11	86.11	88.89	91.67
12	83.33	80.56	83.33
13	86.11	80.56	72.22
Mean	83.33 ± 8.56	82.48 ± 10.17	85.26 ± 9.31

**Table 3:** HbR-based binary classification accuracies between negative emotion and each positive emotion cluster.

Participant	Accuracy (%)		
	Negative vs. Cluster 1	Negative vs. Cluster 2	Negative vs. Cluster 3
1	80.56	77.78	80.56
2	63.89	91.67	75.00
3	97.22	86.11	72.22
4	75.00	77.78	66.67
5	83.33	83.33	80.56
6	86.11	69.44	63.89
7	91.67	75.00	91.67
8	69.44	77.78	80.56
9	75	80.56	63.89
10	91.67	91.67	91.67
11	86.11	86.11	94.44
12	72.22	69.44	66.67
13	72.22	77.78	66.67
Mean	80.34 ± 9.98	80.34 ± 7.21	76.50 ± 11.03

The HbO-based binary classification accuracies between positive emotion clusters are shown in Table 4. Individual-level binary classifications among them achieved an averaged accuracy of 73.79 ± 11.49% (77.56 ± 7.39% for encouragement vs. playfulness, 73.29 ± 11.87% for playfulness vs. harmony, 70.51 ± 13.96% for encouragement vs. harmony; The chance levels determined by permutation tests were 49.55 ± 13.32, 48.64 ± 13.05, 48.87 ± 13.10%, respectively). The HbR-based results are shown in Table 5. Individual-level binary classifications among them achieved an averaged accuracy of 66.74 ± 13.04% (74.57 ± 9.94% for encouragement vs. playfulness, 64.53 ± 12.94% for playfulness vs. harmony, 61.11 ± 12.88% for encouragement vs. harmony; The chance levels determined by permutation tests were 48.50 ± 12.53, 48.41 ± 11.97, 47.68 ± 12.30%, respectively).

**Table 4:** HbO-based binary classification accuracies within three positive emotion clusters.

Participant	Accuracy (%)		
	Cluster 1 vs. Cluster 2	Cluster 2 vs. Cluster3	Cluster 1 vs. Cluster 3
1	75.00	63.89	66.67
2	75.00	86.11	75.00
3	83.33	69.44	77.78
4	83.33	55.56	86.11
5	80.56	69.44	86.11
6	77.78	66.67	50.00
7	88.89	86.11	80.56
8	66.67	69.44	58.33
9	83.33	80.56	58.33
10	83.33	88.89	75.00
11	69.44	83.33	72.22
12	77.78	80.56	86.11
13	63.89	52.78	44.44
Mean	77.56 ± 7.39	73.29 ± 11.87	70.51 ± 13.96

**Table 5:** HbR-based binary classification accuracies within three positive emotion clusters.

Participant	Accuracy (%)		
	Cluster 1 vs. Cluster 2	Cluster 2 vs. Cluster3	Cluster 1 vs. Cluster 3
1	58.33	47.22	50.00
2	77.78	77.78	66.67
3	91.67	63.89	72.22
4	80.56	41.67	61.11
5	75.00	63.89	80.56
6	75.00	55.56	58.33
7	88.89	75.00	72.22
8	77.78	77.78	41.67
9	66.67	66.67	38.89
10	80.56	80.56	63.89
11	66.67	72.22	75.00
12	69.44	69.44	63.89
13	61.11	47.22	50.00
Mean	74.57 ± 9.94	64.53 ± 12.94	61.11 ± 12.88

## Discussion

The current study investigated the brain hemodynamic responses to different positive emotions using fNIRS. In line with our previous EEG study (Hu et al., 2017), considerable similarity among the 10 positive emotions induced in the experiment was evidenced by the participants’ subjective reports, leading to three representative positive emotion clusters (encouragement, playfulness, and harmony). The three positive emotion clusters showed different hemodynamic responding patterns, and the HbO-based binary classification between the three clusters achieved an averaged accuracy of 73.79 ± 11.49%, suggesting possible distinct underlying neural mechanisms of the three positive emotion clusters. To the best of our knowledge, this is the first piece of fNIRS evidence demonstrating the differentiation of subdivided positive emotions.

Previous fNIRS findings on the activation in the PFC to the general-level positive emotion have been mixed. One study used image stimuli from the International Affective Picture System (IAPS) to induce positive/negative/neutral emotion states, and found positive emotion was associated with decreased HbO in the left dorsolateral PFC, while negative emotion was accompanied by increased HbO in the bilateral ventrolateral PFCs (Hoshi et al., 2011). Yet another IAPS based study found lower HbO responses in the right PFC to positive emotion than negative emotion (Balconi et al., 2015). Studies with other emotion manipulation methods also revealed inconsistent findings: one study selected visual art to elicit positive and negative emotions, and reported increased oxygenation (HbO-HbR) in medial rostral PFC for positive emotion compared to negative states (Kreplin and Fairclough, 2013), while another study introduced participates to anticipate positive/negative/neutral emotion, and found activation in left dorsal lateral PFC was higher for positive emotion anticipation than negative and neutral conditions (Wang et al., 2018). In the present study, we found a mild correlation between the general emotion valence and lateral HbO activations, which suggests higher HbO responses in the lateral PFC were associated with more positive emotions. Although we cannot make further inference due to different experimental paradigms, these findings nevertheless indicated the importance of PFC in positive emotion processing.

When we examined the neural responses to the three positive emotion clusters respectively, distinct hemodynamic activation patterns were observed: “Encouragement” emotions were associated with HbO increase in the medial prefrontal area; “Playfulness” emotions reduced the HbR responses in the whole prefrontal area; “Harmony” emotions activated a salient left lateral prefrontal HbR responses. The increased HbO in the medial prefrontal area during “encouragement” emotions may be attributed to the activation of the medial prefrontal area associated with self-relevant processing, as these emotions (pride, inspiration, hope, gratitude, awe) require more self-relevant processing than the other positive emotions (e.g., Tangney, 1999; Van Cappellen and Rimé, 2013). Whereas our stimuli for “harmony” emotions contained film clips about maternal love, the association between maternal love and increased HbO in the right prefrontal area found by Nishitani et al. (2011) did not replicate in the present study. This may be because that the conclusion in Nishitani et al. (2011) was exclusive to mothers with new-born babies, but the participants in our study are all non-mother students. Another alternative explanation is that our stimuli contained not only maternal love but also romantic love, and further comparisons could be conducted between more specific kinds of love in future studies. While there are more distinct hemodynamic patterns for the three positive emotion clusters (such as the decreased HbR associated with “playfulness” emotions), more studies are necessary before providing further interpretation for these findings. In addition, the peak correlation coefficients between HbO/HbR and the three positive emotion cluster scores (0.11, 0.15, and 0.13 for the three cases, respectively) were larger than those between HbO/HbR and emotion valence (peak value of 0.09, as depicted by the colors in Figure 4), further arguing against the conventional view of treating positive emotions as a homogenous group. It should be noted that these correlation results were non-significant and therefore mainly for a descriptive purpose. The non-significance of these results might be due to the small sample size, or a possible high inter-participant variability of the positive emotion responses (e.g., Hamann and Canli, 2004; Kehoe et al., 2011). Further studies are necessary to localize the responsive regions of different positive emotions.

More importantly, the hemodynamic signal based classification results confirmed the separability of these specific positive emotion clusters (average accuracy for paired classifications between the three clusters achieved 73.79 ± 11.49% for HbO and 66.74 ± 13.04% for HbR). The HbO-based classifications in general showed better performance than HbR-based ones, which could be explained by the overall more reliable measurement of cerebral blood flow by HbO than HbR (Malonek et al., 1997; Strangman et al., 2002). These binary classification accuracies were lower than results obtained for positive vs. negative classifications (73.79 ± 11.49 vs. 83.69 ± 9.19% for HbO and 66.74 ± 13.04 vs. 79.06 ± 9.47% for HbR), suggesting one positive emotion is more similar to other positive emotions than negative ones. Nevertheless, the discriminability between the three positive emotion clusters was still well above chance level. In addition, the classifications were performed on an individual level at the time scale of 10 s without any artifact rejection procedures. While the neural separability between different positive emotion categories might be underestimated, these results offered direct support for the potential practical real-time emotion recognition applications.

It is worthwhile to note that a machine learning approach was employed in the present study. Here we mainly focused on single-participant-level classification results to reflect the separability of the neural responses to different positive emotions. While the sample size is smaller than typical neuroscience studies that have usually focused on group-level statistics, it is comparable with existing fNIRS based affective computing studies using machine learning methods (e.g., Tai and Chau, 2009; Moghimi et al., 2012; Heger et al., 2013; Aranyi et al., 2016). Moreover, our results for positive vs. negative classifications were in a similar range as these previous studies (i.e., 70∼95%) and most participants in the present study got classification results well above chance level (for the classifications between negative and positive emotions, mainly in the range of 70∼95%; for the classifications within three positive clusters, mainly in the range of 65∼85%; with one exception of the participant #13 showing chance-level performance for two out of three binary classifications), supporting the validity of our conclusion. Nevertheless, the machine learning approach is limited in its explanatory capacity toward mechanism interpretations (Shmueli, 2010), further studies with a larger sample size would help to gain more insights about underlying neural mechanisms of different positive emotions.

Admittedly, several limitations of the present study should be noted. First, the selection of the 10 kinds of positive emotions was supposed to include the most frequently experienced positive emotions in people’s daily life (Fredrickson, 2013), rather than cover all the possible positive emotions. Accordingly, the three clusters based on these 10 positive emotions could not be expected to explain all the variants of positive emotions. Second, as mentioned above, different emotion-eliciting paradigms might lead to different conclusions. Future studies should be conducted with more kinds of emotion stimuli (such as images and sound), and this also calls for more standardized databases for emotion stimuli of different positive emotions (e.g., Ge et al., 2018). Third, data analyses in this study were based on the most basic HbO and HbR changes, not including more complex features such as slope, skewness, and kurtosis of HbO and HbR signals. Therefore, more feature extraction methods together with advanced machine learning techniques could be explored in future studies. Last but not least, due to the limited film clip materials and the limited fNIRS channel coverage, our results do not necessarily indicate that there are only three prominent positive emotion clusters, but rather suggest that at least three clusters could be well-separated based on human hemodynamic responses over the frontal regions.

The differentiation of positive emotions is not only of theoretical importance, but also of practical value. For example, the differentiation of specific positive emotion experience is highly valued in the field of affective product design and interaction, because user’s emotion experiences to products are more nuanced than what is captured with a general bipolar dimension of valance (Yoon et al., 2016). Therefore, positive emotion evaluations with higher granularity are expected to be beneficial in product design process, and provide users with more fitting positive experiences. In the field of consumer decision-making, recent studies have also stressed that emotions of the same positive valence would have different effects on consumers’ product preferences and decision-making behaviors (Griskevicius et al., 2010; Winterich and Haws, 2011), which indicated the significance of distinguishing different positive emotions in marketing strategy making. The plausibility of differentiating specific positive emotions has been supported by the present study at the neural level. Together with fNIRS’s high portability, low running cost and the relative robustness against motion and electrical artifacts, our findings also suggested the potential of implementing a more fine-grained positive emotion recognition system with the fNIRS technique.

## Ethics Statement

This study was carried out in accordance with the recommendations of Tsinghua University Research Ethics Committee in Psychology Department with written informed consent from all subjects. All subjects gave written informed consent in accordance with the Declaration of Helsinki. The protocol was approved by Tsinghua University Research Ethics Committee in Psychology Department.

## Author Contributions

XH contributed to the conception design, data analysis, and drafting the work. CZ contributed to data collection and analysis. FW, Y-JL, and C-HI contributed to revising the work. DZ contributed to the conception design, data interpretation and drafting, revising the work. All authors approved the work for publication.

## Conflict of Interest Statement

The authors declare that the research was conducted in the absence of any commercial or financial relationships that could be construed as a potential conflict of interest.

## Supplementary Material

TABLE S1 Basic information of the materials.
